# Enhanced endoscopic papilla visualization for endoscopic retrograde cholangiopancreatography under indocyanine green: proof of concept in a preclinical model

**DOI:** 10.1016/j.vgie.2026.04.050

**Published:** 2026-05-06

**Authors:** Ariosto Hernández-Lara, Alain García, María Rita Rodríguez-Luna, Kohei Mishima, Fanélie Wanert, Santiago Philibert-Rosas, Lee Swanström, Silvana Perretta

**Affiliations:** 1 Institut de Chirurgie Guidée Par l'image (IHU), Strasbourg, France; 2 Institut de Recherche Contre les Cancers de l'appareil Digestif (IRCAD), Strasbourg, France; 3 University of Wisconsin–Madison, Madison, Wisconsin, United States

## Abstract

**Background and Aims:**

ERCP is technically demanding, often requiring up to 300 procedures for proficiency. Indocyanine green (ICG) has emerged as a promising tool for enhancing biliary structures. This study aimed to evaluate the feasibility of ICG-based near-infrared (NIR) imaging for improved papillary orifice localization and cannulation in a porcine model.

**Methods:**

Four 30-kg pigs underwent laparotomy and duodenoscopy with a therapeutic colonoscope. Scopolamine (0.5 mg/2 mL) was administered, followed by 0.1 mg/kg of ICG administered as an intravenous bolus and a 20-mL saline flush. An NIR-enabled laparoscope was introduced through an enterotomy, enabling concurrent endoscopic and laparoscopic assessment of the duodenal papilla. Standard ERCP tools were used for cannulation, and a precut technique was used when standard methods failed.

**Results:**

ICG-enhanced bile was observed at approximately 5 minutes. Although the dye spread diffusely by 10 minutes, the papilla remained distinguishable, and fluorescence at the papillary orifice improved localization to guide targeted cannulation and precut orientation.

**Conclusions:**

ICG-enhanced NIR imaging can facilitate papillary identification in a porcine model, suggesting potential applicability as an adjunct when the papilla or bile duct orifice is difficult to identify. This approach is currently limited by the need for NIR-capable imaging platforms and the lack of commercially available NIR-enabled duodenoscopes.

## Introduction

ERCP is a transoral procedure used to perform therapies in pathologies involving the bile duct, including obstructive and nonobstructive indications. The method requires accuracy and a high level of technical competence. Endoscopists need to execute up to 300 ERCPs before they can perform this procedure with acceptable success rates.^1^, ^2^, ^3^

In most ERCPs, the papilla is readily identifiable; however, in selected cases (periampullary diverticulum, edema, inflammation, or distorted anatomy), localizing the papillary orifice can be challenging and may delay biliary access. Novel advances in biliary surgery include the use of indocyanine green (ICG), which has proven to be a better visualization of biliary anatomy to prevent adverse events under different validated doses^4^^,^^5^ (Fig. 1). ICG-infrared technology has been applied in flexible endoscopy but is not integrated into duodenoscopes.^6^^,^^7^ In selected cases, localization of the papillary orifice can be challenging and may delay biliary access.

**Figure 1:**
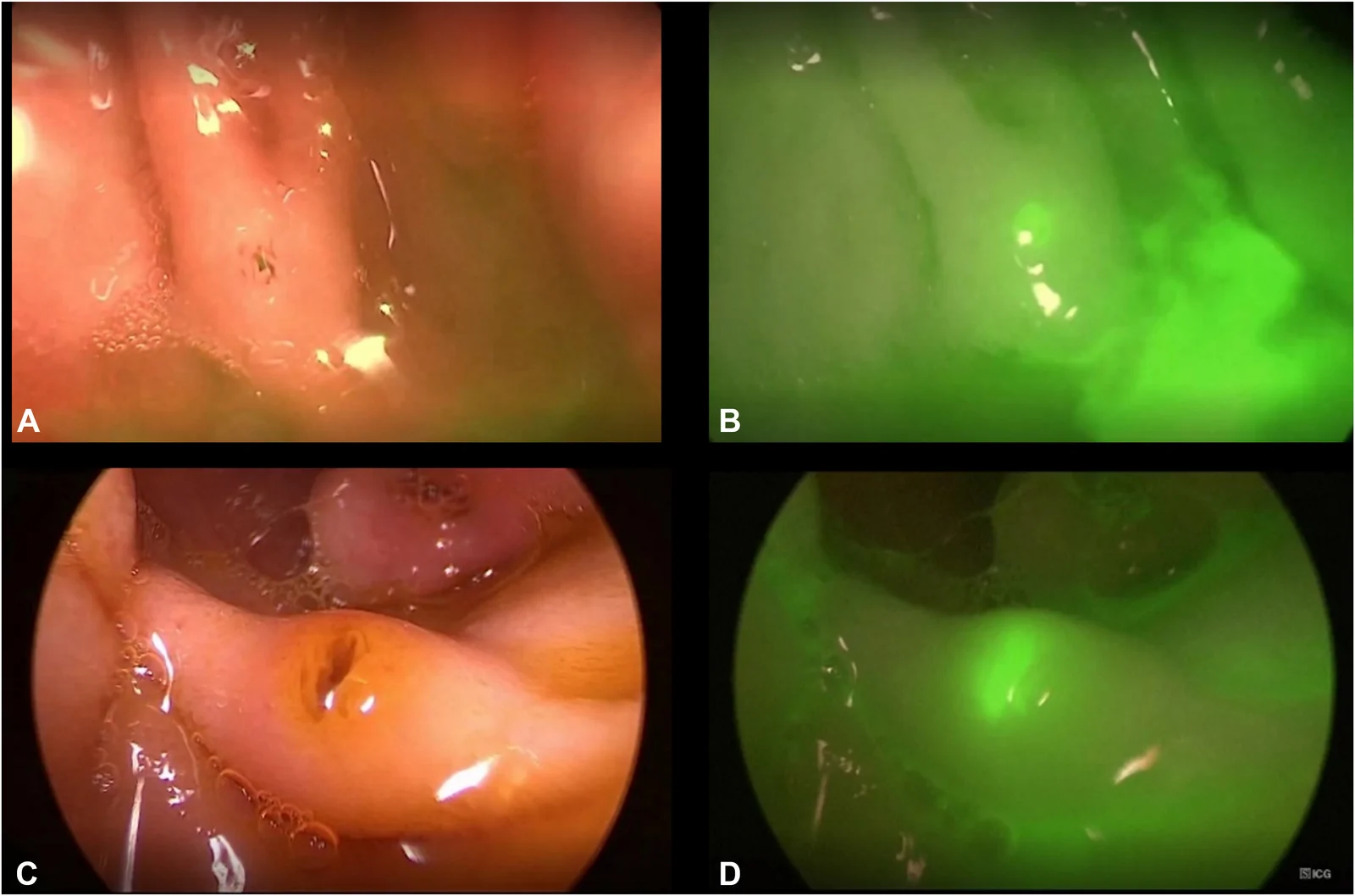
ICG/NIR localization overtime. **A,** White-light view of the papillary region approximately 5 minutes after IV ICG. **B,** NIR view approximately 5 minutes after IV ICG showing ICG-enhanced bile outflow at the orifice. **C,** White-light view 1 hour after IV ICG. **D,** NIR view 1 hour after IV ICG showing increased background fluorescence with the papillary region still identifiable. *ICG*, Indocyanine green; *IV*, intravenous; *NIR*, near-infrared.

## Case

### Procedure description

With four 30-kg average secondary pigs used for endoscopy training purposes other than biliopancreatic procedures (Fig. 2) under deep sedation, we performed a cooperative procedure in a prone position in between a laparotomy and duodenoscopy to visualize the duodenal papilla (Video 1, available online at www.videogie.org).

**Figure 2:**
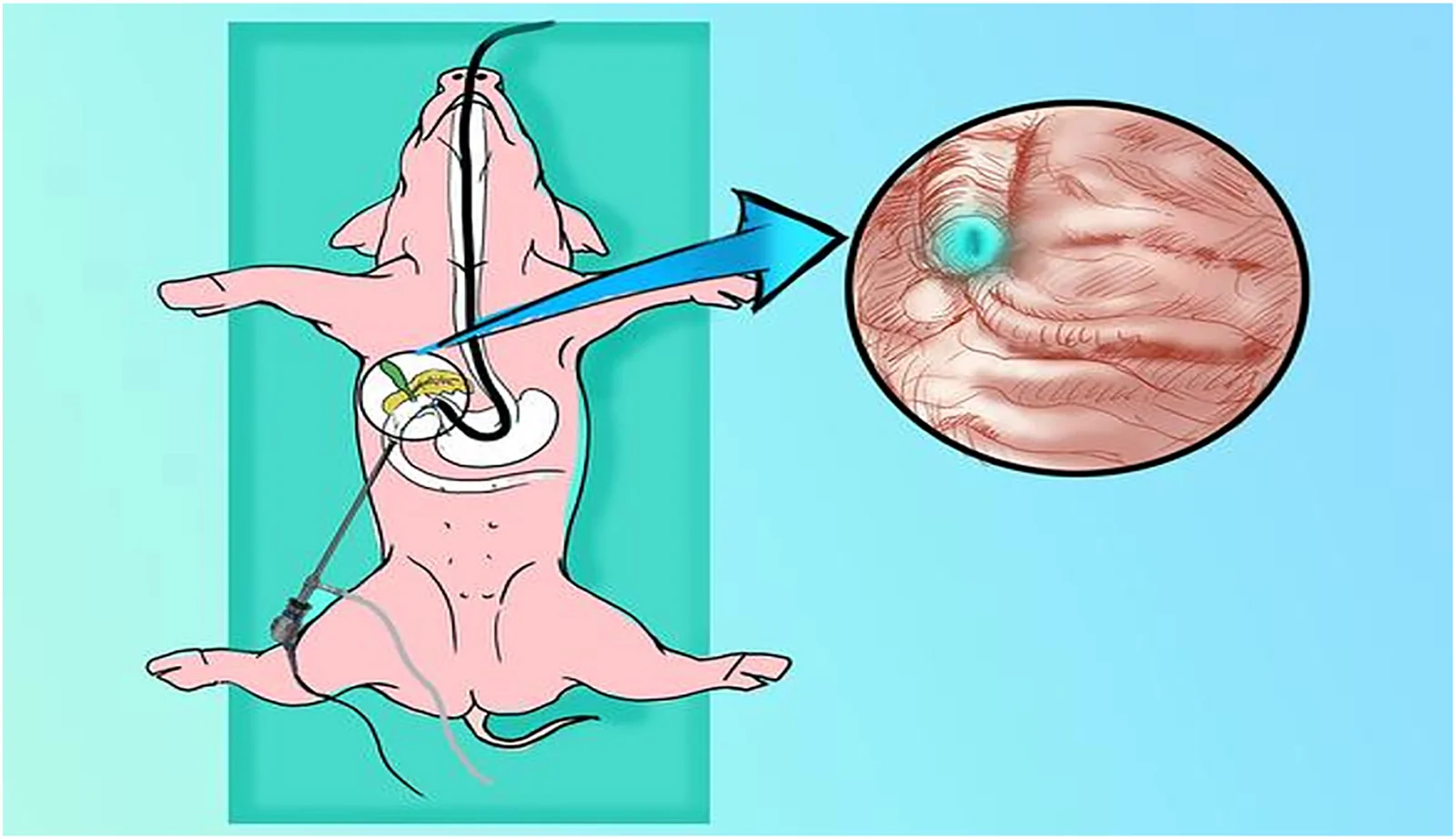
Schematic of the porcine set-up showing transoral duodenoscopy and intraduodenal near-infrared laparoscopy via enterotomy, with an inset highlighting the papillary region.

At the beginning of the procedure, we injected 1 ampulla of scopolamine (0.5 mg/2 mL) as an antispasmodic to reduce duodenal peristalsis and stabilize the endoscopic view and 0.1 mg/kg of ICG, administered as an intravenous bolus via a peripheral venous access. According to the manufacturer's specifications, we diluted the ICG powder in a 5% glucose solution to a final dilution of 25 mg/10 mL. After ICG injection, a 20-mL bolus of saline solution (0.9% sodium chloride) was administered to ensure that the ICG reached the systemic venous blood. The decision to use scopolamine was based on local availability and routine use as an antispasmodic in our unit; glucagon is an alternative antispasmodic that may be used in other centers to reduce duodenal peristalsis.

First, we performed a duodenoscopy with a therapeutic colonoscope (EC34-i10; Pentax, Tokyo, Japan) using the narrow-band image (i‑SCAN 3) feature already integrated to visualize the papilla (Fig. 3). Because a forward-view therapeutic colonoscope lacks the side-view orientation and elevator mechanics of a standard duodenoscope, aligning accessories with the biliary axis was suboptimal and contributed to initial cannulation difficulty.

**Figure 3:**
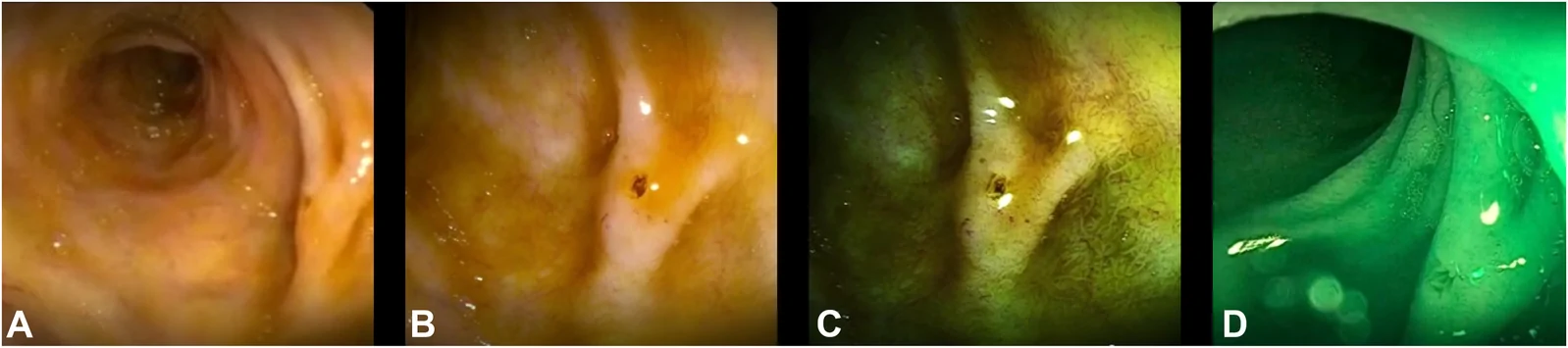
Duodenoscopy. **A,** Duodenal lumen view. **B,** Papilla identified under standard white light. **C,** Papilla under image-enhanced endoscopic mode. **D,** Additional image-enhanced view demonstrating the papillary region.

After endoscopic visualization of the papilla, the tip of a near-infrared (NIR)/ICG IMAGE1 S laparoscope (Karl Storz, Tägerwilen, Switzerland) was introduced into the anterior wall of the duodenum through an enterotomy 4 cm away from the area of the papilla under endoscopic guidance and transillumination (Fig. 4). A reference ICG card (Green Balance; Diagnostic Green, Aschheim-Dornach, Germany) was placed near the region of interest to compute the normalized fluorescence afterward.

**Figure 4:**
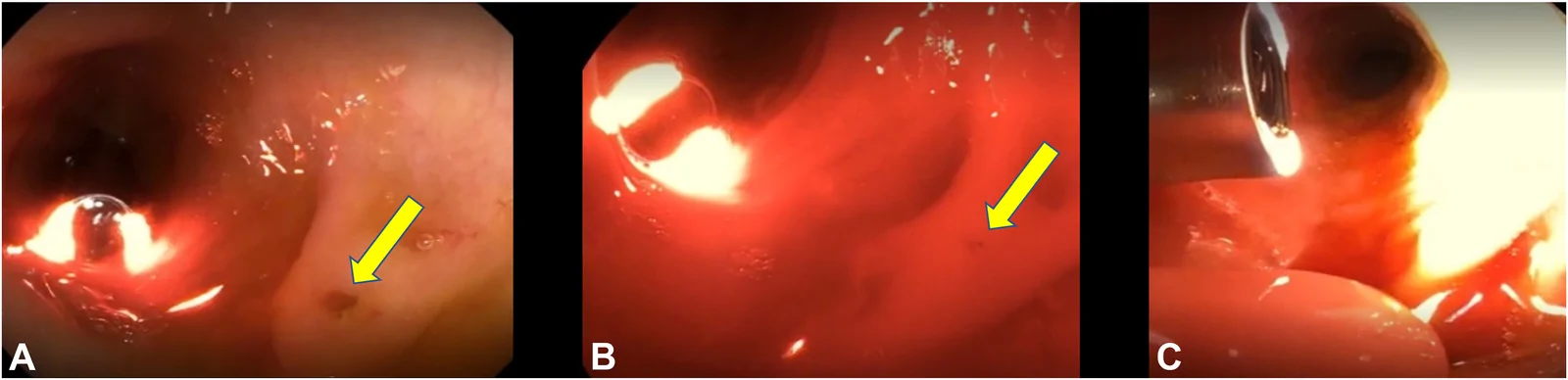
Enterotomy plus intraduodenal positioning of the laparoscope. **A,** Endoscopic view showing the papilla (*arrow*). **B,** Endoscopic view during positioning for the enterotomy site near the papilla (*arrow*). **C,** Laparoscope introduced through the enterotomy into the duodenum (operative view).

The images were captured using the laparoscopic camera, which has a standard white-light observation mode that can be switched to an NIR mode using a footswitch pedal, which simultaneously activates the NIR light source and a bypass filter.

Both laparoscope and colonoscope were used to locate the duodenal papilla. The position of the scopes was then stabilized and documented (Fig. 5).

**Figure 5:**
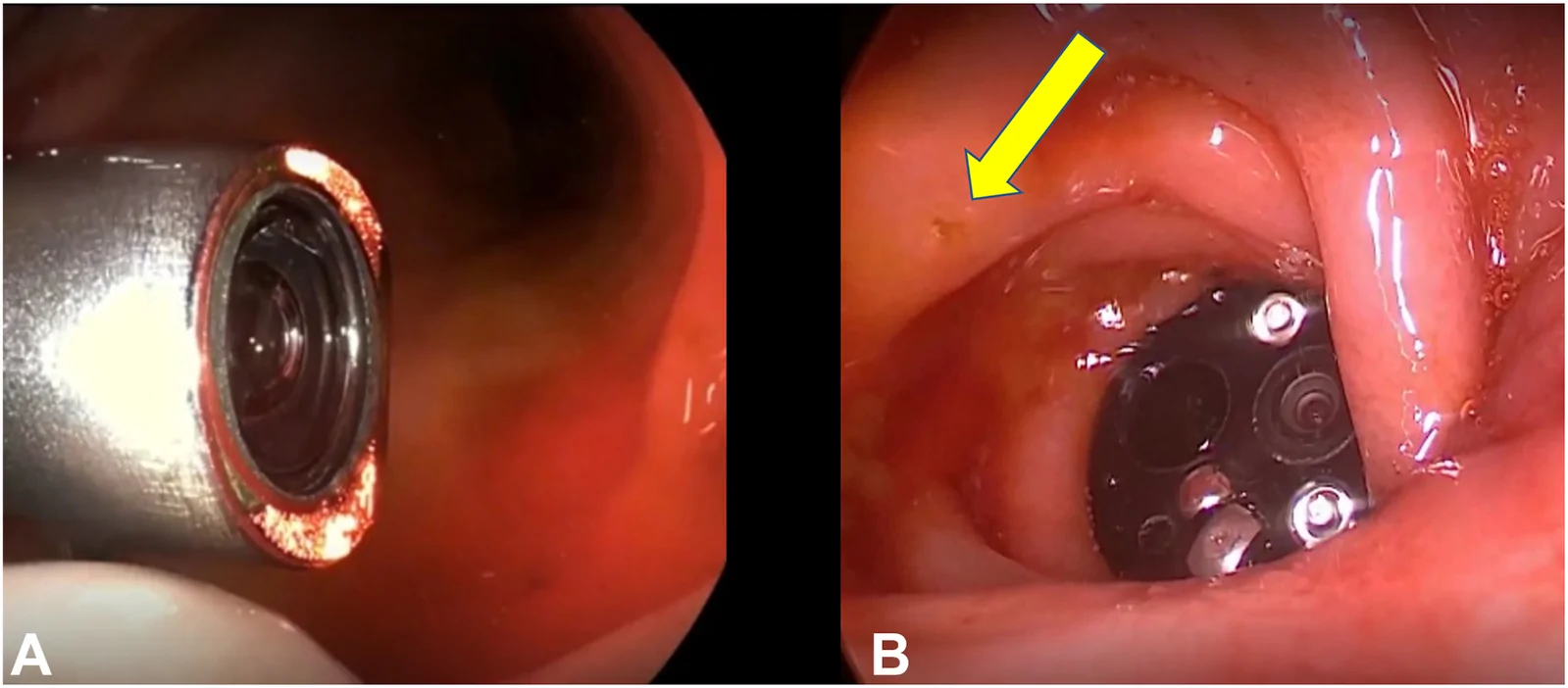
Rendezvous. **A,** Laparoscope tip visualized within the duodenal lumen. **B,** Endoscopic view demonstrating both scopes positioned in the duodenum relative to the papilla (*arrow*).

After approximately 5 minutes from the injection of ICG, we documented the exit of bile from the papilla enhanced with ICG under infrared exposure with the laparoscope. This ICG-enhanced bile egress highlighted the papillary orifice under NIR and improved localization to guide subsequent cannulation maneuvers (Fig. 1).

While injecting the ICG dye, we waited for 10 minutes and observed the area of the papilla under the infrared spectrum (Fig. 6). We found that the ICG-enhanced bile was spread all over the surgical field, making it difficult to accurately determine the area of interest.

**Figure 6:**
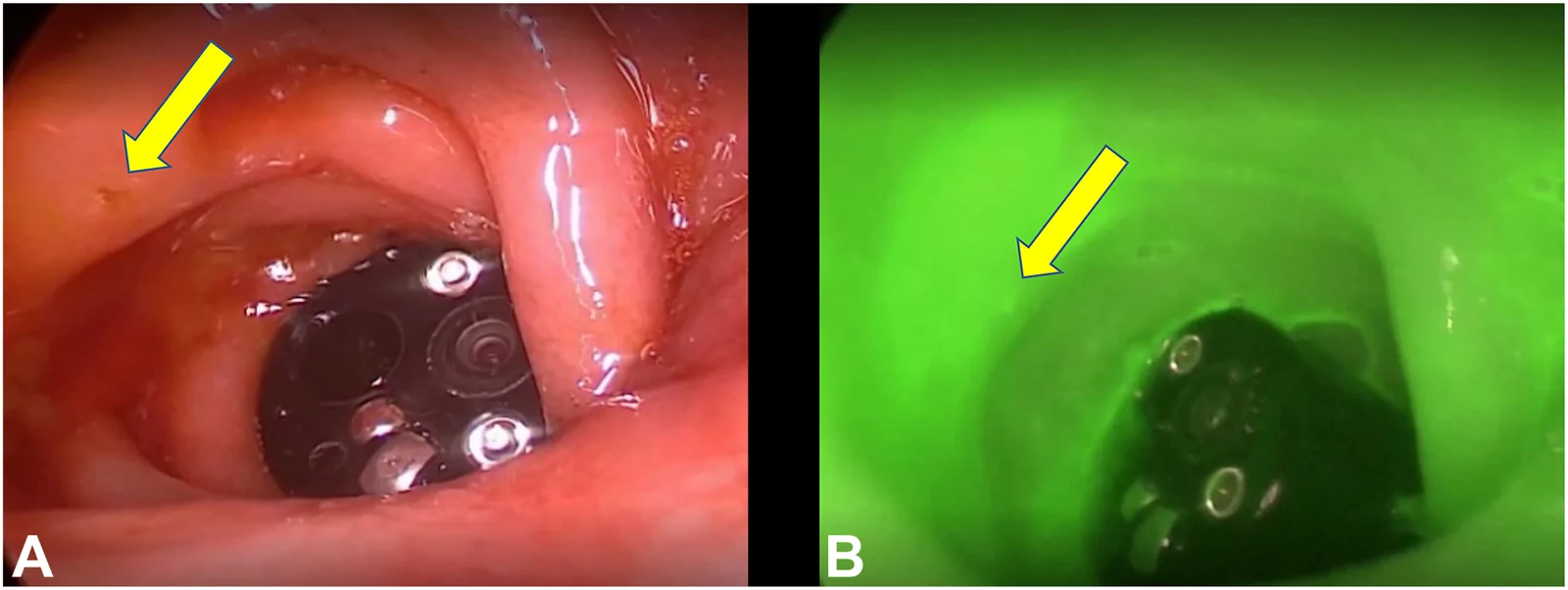
Papillary region seen from enterotomy. **A,** Endoscopic view of the papillary region/orifice (*arrow*). **B,** Corresponding near-infrared view from the enterotomy site highlighting the region (*arrow*).

However, we were able to compare the area of the papilla that stood out from the rest of the surgical field at different times. Under infrared laparoscopic view, a sphincterotomy cannulation was attempted using conventional ERCP tools (DomeTip sphincterotome, Cook Medical, Bloomington, Ind, USA; 0.035-inch Acrobat guidewire, Cook) through the working channel of the therapeutic colonoscope (3.2 mm). After several attempts due to the difficult position and limited view of the instruments, we proceed with an advanced cannulation technique with a precut (Fig. 7).

**Figure 7:**
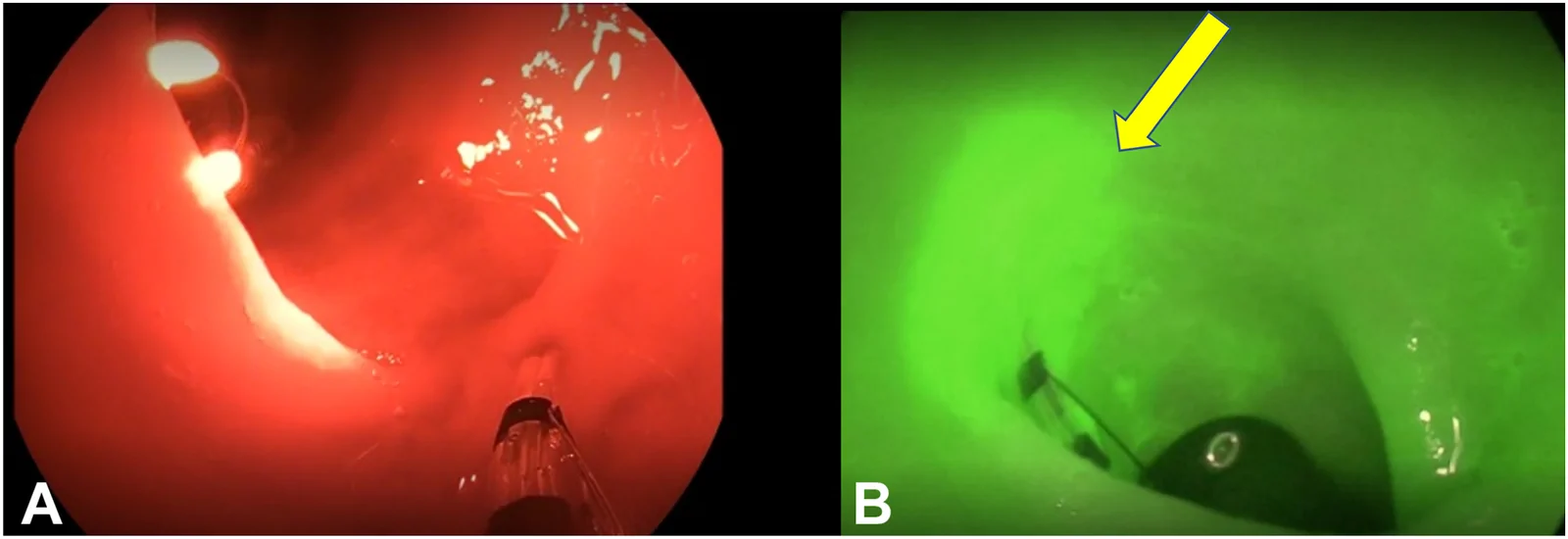
Cannulation attempt under near-infrared (NIR) guidance. **A,** Endoscopic view during cannulation attempt with a sphincterotome. **B,** Corresponding NIR view during cannulation attempt (*arrow* marking the target region).

Using pure cut energy (30 W), we made an incision in the papilla with the precut knife (Fusion Needle Knife; Cook) cutting toward the upper edge of the papilla by approximately 2 mm (Fig. 8) and then advanced the sphincterotomy again into the bile duct under direct visualization and stereotactic confirmation by the team in charge of the laparotomy.

**Figure 8:**
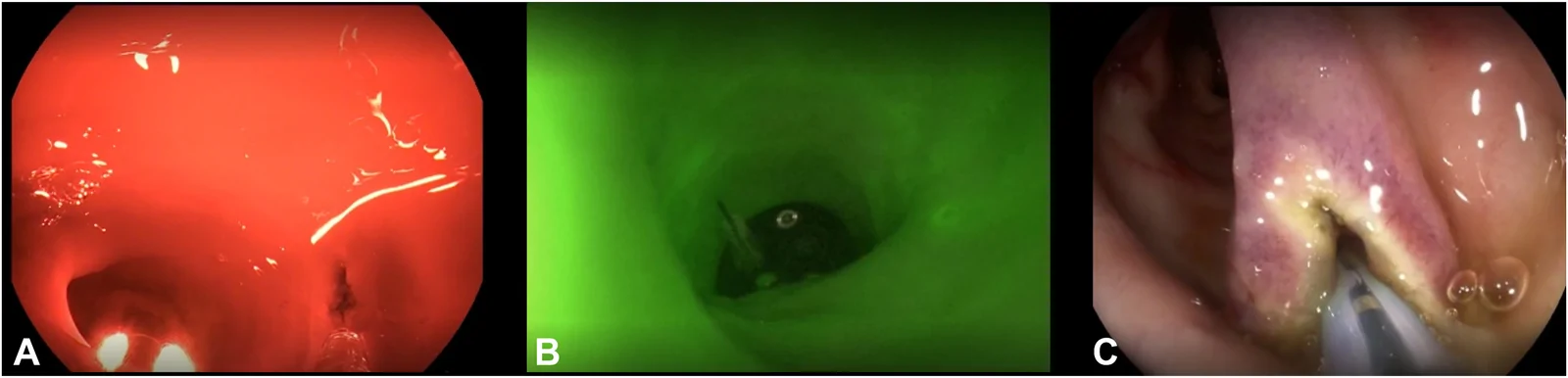
Precut and cannulation. **A,** Needle-knife precut step under endoscopic visualization. **B,** Near-infrared view after precut showing the target region under fluorescence guidance. **C,** Close-up confirming cannulation at the papilla.

## Discussion

ICG-enhanced NIR imaging functioned primarily as a localization adjunct rather than a substitute for a cannulation technique. In this porcine model, ICG was rapidly excreted into bile, and fluorescence at the papillary orifice improved identification of the target region by approximately 5 minutes. However, by approximately 10 minutes, diffuse spread of ICG-enhanced bile increased background fluorescence, suggesting that dose and timing may affect contrast and should be optimized for clinical translation. Swine-versus-human pancreatobiliary anatomic differences may influence ICG signal at the papilla. In pigs, biliary drainage occurs at the major papilla near the pylorus with separate pancreatic drainage at a more distal minor papilla,^8^^,^^9^ whereas in humans, the common bile duct and pancreatic duct commonly converge at the major papilla, with variable anatomy.^10^ Although ICG dosing regimens have been described in humans^4^, ^5^, ^6^ for biliary fluorescence imaging, differences in biliary kinetics between porcine models and humans may alter the optimal timing window.

Other adjuncts that may facilitate orifice localization include cholecystokinin analogs such as sincalide, which can increase bile outflow and help localize the papilla.^11^ A key limitation is that this technique depends on NIR-capable imaging, which is not routinely available during standard ERCP, and NIR-enabled duodenoscopes are not widely available. A practical human application would likely require either development and evaluation of NIR-enabled duodenoscopes, or use in laparoscopy-assisted rendezvous settings where an NIR laparoscope is already available. If NIR-enabling technology were integrated into duodenoscopes, ICG could become a useful adjunct in cases where the bile duct orifice is difficult to localize. ICG is generally considered low cost per case, but pricing varies by region.^12^

## Disclosure

The following author disclosed financial relationships: L. Swanström: Consultant for Boston Scientific, Magnapath, Olympus, Quelon, Taurus Endoscopy, Magnamosis, Aponos, Virtual Port, Covidien, Auris, Mediglobe, Endogastric Solutions, Titan, Stryker, Fractyl, Apollo Endosurgery, Human Extensions, and Transenteryx. All other authors disclosed no financial relationships.

## Supplementary data

Video 1 Enhanced endoscopic papilla visualization ERCP.
